# Comment on “Intraocular fluid biomarkers (liquid biopsy) in human diabetic retinopathy” Graefes Arch Clin Exp Ophthalmol. 2021 Jul 3. doi: 10.1007/s00417-021-05285-y

**DOI:** 10.1007/s00417-021-05462-z

**Published:** 2021-10-30

**Authors:** Diana Anna Dmuchowska, Karolina Pietrowska, Adam Kretowski, Michal Ciborowski

**Affiliations:** 1 Department of Ophthalmology, Medical University of Bialystok, 24a M. Sklodowskiej-Curie St. 15-276, Bialystok, Poland; 2 Metabolomics Laboratory, Clinical Research Centre, Medical University of Bialystok, Bialystok, Poland; 3 Department of Endocrinology, Diabetology and Internal Medicine, Medical University of Bialystok, Bialystok, Poland

Dear Editor,

We have read with great interest the paper by Midena E. et al. entitled “Intraocular fluid biomarkers (liquid biopsy) in human diabetic retinopathy” [1]. The authors reviewed proteomic and metabolomic studies of aqueous (AH) and vitreous humor (VH) of patients with diabetes mellitus. As the metabolomics of AH is within the scope of our interests and experience, we would like to comment on various aspects discussed in this paper. Our comments, addressing both analytical and clinical aspects of the problem in question, might be helpful for researchers planning future studies involving patients with diabetes.

According to Midena et al., various stages of eye disease should be taken into account during the research on diabetic patients. The authors suggested a tailored approach based on phenotypic characteristics of eye disease. We postulate to expand this idea even further, so not only the ocular pathology but also characteristics of diabetes are considered. The list of characteristics that could be studied includes type and stage of diabetes, level of its control (e.g. expressed by glycemia and levels of hemoglobin A1c, insulin or C-peptide), and kind of anti-diabetic treatment. All these factors might potentially affect metabolomic profiles of AH and VH, but to the best of our knowledge, they were not considered in any of the studies conducted thus far.

Furthermore, Midena et al. suggested implementing quite a novel concept according to which biochemical changes are correlated with imaging features to support the detection of biochemically proven structural biomarkers. In this context, we suggest also evaluating the choroid, the structure which is not easily available for metabolomics studies. A potential role of diabetic choroidopathy in the pathogenesis of diabetic retinopathy and diabetic macular edema cannot be neglected, as the choroid provides blood to the outer retina. Furthermore, it is the ciliary body that produces the AH and forms the uveal tract together with the iris and choroid. We showed unparallel involvement of retinal and choroidal circulation in patients with diabetes, which might reflect a multifaceted impact of the disease on the two blood supply systems [2].

Regarding the analytical aspects, the valuable concept of comprehensive liquid biopsy of AH and VH suggested by Midena et al. might be expanded using a multiplatform approach. We explained this idea in our publication, using metabolomic analysis of the AH of patients with diabetes as an example [3]. A number of various analytical platforms are suitable for metabolomic studies. The most commonly used methods include nuclear magnetic resonance (NMR) or mass spectrometry (MS) coupled with various separation techniques, such as liquid chromatography (LC–MS), gas chromatography (GC–MS), and capillary electrophoresis (CE-MS). Also, the sample preparation procedure [4] and type of analysis (targeted/untargeted) can make a difference. Each of the methods mentioned above is suitable for detecting different metabolite classes, and none of them provides information about the entire metabolome. Consequently, a multiplatform approach would enable us to detect a broader spectrum of metabolites. Then, a multi-omic approach seems an attractive idea in a further perspective. Moreover, given that AH originates from the blood, the analysis of not only AH and VH but also the blood from the same patients could be a reasonable approach.

Finally, Midena et al. comprehensively reviewed various pathogenetic processes, including mitochondrial dysfunction and oxidative stress, involved in the AH of patients with diabetes. Their results are consistent with our findings in diabetic patients [5]. Interestingly, we observed a decrease in the level of tryptophan-derived metabolites that act as UV filters and discussed the potential role of this phenomenon in earlier and faster development of cataract in patients with diabetes. Moreover, we found metabolites related to gut microflora in the AH and were the first to report the presence of glycosylated amino acids in this biological material.

In summary, along with Midena et al., we suggest a broader approach considering both clinical (e.g. the stage of diabetes and eye disease, imaging findings) and analytical (liquid biopsy, multiplatform metabolomics, and multi-omic approaches) aspects during the design of future metabolomic studies on diabetic eye diseases.
